# Magnetic Resonance Imaging and Interictal Electroencephalography Findings in Newly Diagnosed Epileptic Children

**DOI:** 10.3390/jcm7060134

**Published:** 2018-06-01

**Authors:** Mehmet Alp Dirik, Burcin Sanlidag

**Affiliations:** 1 Faculty of Medicine, Department of Radioloy, Suat Gunsel University, Kyrenia 99138, North Cyprus; mehdirik@gmail.com; 2 Faculty of Medicine, Department of Pediatrics Division of Pediatric Neurology, Near East University, Nicosia 99138, North Cyprus

**Keywords:** magnetic resonance imaging, electroencephalography, epilepsy, childhood

## Abstract

Introduction: Epilepsy is one of the most frequently diagnosed chronic neurological disorders in children. Diagnosis is often based on seizure history and electroencephalography (EEG) assessment. Magnetic resonance imaging (MRI) is recommended for etiologic workup and intervention requirements. We aimed to detect by MRI if focal structural abnormalities are present in the brain in relation to interictal epileptiform discharges (IED). Material and Methods: The study was designed retrospectively. The data were collected from patients admitted to Near East University, Department of Pediatric Neurology, who were aged between 3 months and 18 years and who were diagnosed with epilepsy. The cases considered in the current study, however, were patients that had an EEG record prior to initiating treatment and an MRI within the first six months following diagnosis. Results: Among 222 patients, 212 (95.5%) had IED, and 92 (41.4%) had abnormal MRI results. The most frequently seen abnormalities detected by MRI were encephalomalacia, hydrocephaly, and atrophy. Among patients who had IED, the ones with multifocal IED were documented to have a statistically significant higher rate of abnormalities in MRI scans. In other patients, IED had no significant correlation with structural lesions detected by MRI. Conclusion: IED can be unrelated to MRI findings. Focal IED were not statistically concordant with the structural lesions detected by MRI. However, for the cases with multifocal discharges revealed by interictal EEG, the rate of abnormalities detected using MRI was 68%. Therefore, the likelihood of detecting abnormalities using MRI in patients with multifocal IED does support the necessity of the use of MRI in early diagnosis stages.

## 1. Introduction

Epilepsy is one of the most important and prevalent neurological disorders among children. The prevalence of epilepsy during the childhood period varies between 3.2 and 5 cases per 1000 children in developed countries and 3.6 and 44 cases per 1000 children in developing countries [1]. Epilepsy also has consequences on the cognitive, psychological, and social functioning of the affected individuals [2,3,4].

The International League Against Epilepsy (ILAE) defines epilepsy as the occurrence of two or more unprovoked seizures apart from each other over 24 h, with a >60% risk of having another seizure in the following 10 years or the development of an epilepsy syndrome [5].

The diagnosis of epilepsy can be based on the occurrence of seizures together with electroencephalography (EEG) recordings. EEG is an important examination method used in the classification and identification of epilepsy syndromes. EEG can also assist in the recommended choice of an antiepileptic drug for a patient. It has been established that antiepileptic drugs should be prescribed on the basis of the seizure pattern revealed through both clinic and EEG findings [6].

EEG findings in patients with bilateral brain lesions were demonstrated to reveal approximately 55% of bilateral interictal discharges (IED). However, in a study, focal IED were found to be unrelated to lesions detected by MRI [7,8].

MRI is not a diagnostic tool for epilepsy. However, it has an important role in the identification of the underlying structural lesions. Diagnosis and management of epilepsy have changed in the last decades through the contribution of MRI. The use of high-quality MRI and the use of epilepsy protocols according to guidelines of the ILAE have improved the sensitivity of neuroimaging for structural lesions [9]. Approximately 20% of patients were shown to have an epilepsy-related pathology by MRI in a large-scale study involving 2000 patients with seizures [10]. Additionally, structural lesions were demonstrated in 82–86% of cases with refractory epilepsy [11,12]. In different studies, the rates of abnormalities detected using MRI in epileptic children were between 28.5% and 55.86% [13,14,15].

Some epileptic lesions can be demonstrated by structural MRI. The common ones include hippocampal sclerosis, epilepsy-associated tumors, focal cortical dysplasias, and encephalitis (limbic encephalitis, Rasmussen encephalitis). MRI is an important diagnostic tool, especially in those cases. It provides etiological and prognostic information [16].

Therefore, structural MRI is a necessary diagnostic technique for cases with epilepsy. The ILAE recommends MRI in cases with localization-related to epilepsy, in cases for which the diagnosis is difficult, in situations where the epilepsy syndrome has a suspected symptomatic cause, and for any suspected epilepsy cases in children younger than 2 years of age [9,17].

In this study, we aimed to assess the relationship between interictal EEG findings before the initiation of an antiepileptic treatment and MRI lesions in children with newly diagnosed epilepsy.

## 2. Material and Methods

The medical history of patients aged between 3 months and 18 years of age was collected retrospectively for a five-year period from Near East University, Faculty of Medicine, Department of Pediatric Neurology.

The study was conducted in accordance with the Declaration of Helsinki, and the protocol was approved by the Ethics Committee of YDU/2018/55-525

EEG was performed interictally by positioning electrodes on the scalp of the patients in accordance with the international 10–20 system [18], Nicolette One. Silver–silver chloride electrodes were used, and the space between the electrodes and the skin was filled with a conductive paste. A total of 16 channeled EEG was performed with a low-pass filter, a high-pass filter, and a notch filter, at 70 Hz, 1 Hz, and 50 Hz, respectively, at a rate of 30 mm/s. At least 30 min of recording were completed both while the patient was asleep and while he/she was awake; the EEG readings were then examined by visual analysis by a pediatric neurologist. If the first EEG record was classified as normal, a second record was taken following activation procedures such as sleep deprivation. For statistical purposes, interictal epileptic discharges (IED) were classified as follows: normal, focal, generalized, multifocal, or first focal to bilateral (primarily focal, then generalized).

Standard MRI scans were taken within a six-months period following diagnosis. MRI examinations were performed using 1.5 Tesla (Siemens) and 3 Tesla (Siemens) unit scanners with a pediatric epilepsy protocol.

The protocol used was composed of: (a) spin echo that was sagittal T1-weighted; (b) fast spin-echo that was axial T2-weighted; (c) coronal oblique fast multiplanar inversion recovery; (d) coronal oblique fast fluid-attenuated inversion recovery; (e) axial diffusion (one shot, spin echo echo-planar), b = 1000 in all directions, (f) axial three-dimensional spoiled gradient-recalled echo. The imaging time took approximately 26 min, and a total of 34 examinations were taken. Scanning by 1.5–3 Tesla with a magnet system was performed. Standard procedures had axial, sagittal, and, more frequently, coronal images, using clinical sequences. These studies were evaluated as adequate or better in scan quality. The most helpful MRI sequences for detecting abnormalities in the brain are T1-weighted and T2-weighted sequences.

MRI was initially performed without contrast material. In case of a suspicious image, gadolinium was administered to demonstrate type and characteristics of the lesion.

For statistical analysis, SPSS version 17 (IBM, Armonk, NY, USA) was used, and the Chi-square test and descriptive statistics were used for evaluation. A *p* < 0.05 was accepted as statistically significant.

## 3. Results

Among 244 patients, 222 had an EEG record prior to antiepileptic treatment and underwent MRI within six months of diagnosis. These 222 patients were selected and enrolled in the study.

This group was composed of 75 (33.9%) females and 147 (66.2%) males. The mean age at the onset of the condition was 48 months.

A total of 212 (95.5%) patients displayed an abnormality in the EEG record, whereas 10 (4.5%) patients had a normal EEG record. Ninety-two (41.4%) patients revealed an abnormality detected using MRI. The findings from the EEG and MRI examinations are displayed in Table 1.

On the basis of the EEG records, 212 of the 222 patients were diagnosed with epileptiform activity.

The EEG and MRI results were both abnormal in 87 (39.2%) patients. In five (2.3%) patients, initial EEG and MRI were normal. In five (2.3%) cases, EEG was classified as normal, and MRI as abnormal. For 125 (56.3%) patients, EEG was classified as abnormal, and MRI as normal.

When the EEG findings were evaluated in relation to the presence of pathology detected by MRI, the *p* value was found to be 0.007, i.e., statistically significant.

The characteristics of the EEG in relation to the presence of imaging abnormalities are reported in Table 2.

In the 212 patients with IED, multifocal IED were found to be related to higher rates of abnormalities in MRI (*p* = 0.004) (Table 3). No statistical significance was detected for other types of IED (*p* > 0.05).

Forty-eight (21.6%) patients had generalized IED and 99 (44.6%) had focal IED. Twenty-five (11.3%) patients had bilateral independent (multifocal) IED. First focal to generalized (secondary generalized) IED was observed in 31 (14.0%) patients.

In the case of focal IED, 19/48 (39.6%) cases had abnormal findings according to MRI, and 29/48 (60.4%) had normal MRI. Of 99 patients who had generalized IED on the basis of the EEG examination, 39 (39.4%) had an abnormal MRI finding, and 60 (60.6%) had normal MRI. In the patients with multifocal IED, the number of abnormalities detected through MRI was 17/25 (68.0%). On the other hand, the number of abnormalities on the basis of MRI was 1/9 (11.1%) in patients with 3 Hz spike–wave discharges, which are characteristic of absence epilepsy. In the cases with first focal to generalized IED, 11 of the 31 (35.5%) patients had an abnormal finding in MRI. Twenty out of 31 patients with first focal to generalized IED had no abnormalities in MRI, as shown in Table 2. EEG discharges in relation to MRI lesions are presented in Table 4.

Encephalomalacia, atrophy, hydrocephaly, and cysts were the most frequently observed pathologies detected in 15 (7.1%), 14 (6.6%), 9 (4.2%), and 9 (4.2%) patients, respectively.

In five cases with normal EEG records, additional abnormal findings based on MRI were detected. The documented MRI lesions also included increased T2 signal intensity in one case, tumor formation in one case, cortical cyst in two cases, and focal encephalomalacia in one case.

In the cases with encephalomalacia, hydrocephaly, gliosis, increased T2 signal intensity, hypomyelinisation, and cyst formation, generalized IED were the most commonly observed pathology on the basis of the EEG records. In the cases with atrophy, focal, generalized, and multifocal IED were observed.

When bilateral brain lesions were evaluated separately, like encephalomalacia, hydrocephaly, atrophy, gliosis, hypomyelination, and increased T2 signal intensity, 27 (49%) out of 55 patients had generalized discharges.

## 4. Discussion

This study aimed to describe the relation between MRI and EEG data in new-onset epilepsy cases.

Among 222 epileptic patients, 212 (95.5%) had documented epileptiform activity based on an EEG examination, and 10 (4.5%) had a normal initial EEG record. Patient’s age, type of epilepsy, time of last seizure, and presence of sleep records affect the presence of IED in routine or prolonged EEG findings [19,20]. The sensitivity of EEG ranges from 10% to 77%. The sensitivity increases to 80–90% in the cases of one or more repeat records, when at least one record is a sleep record. Other than sleep deprivation and sleep records, commonly used activation techniques are hyperventilation (HPV) and intermittent photic stimulation (IPS). HPV and IPS are more effective in inducing generalized IED [21]. The high rate of abnormal EEG (95.5%) in this study is attributed to the fact that a second record was obtained in case of a normal EEG and activation techniques were used.

In a study, nearly half of the localization-related new-onset epilepsy patients had abnormalities on imaging [17], and 15–20% supplied additional useful information regarding the etiological factor of the seizures. Additionally, only 2–4% of them supplied data suggesting urgent medical management [8,22,23,24]. In the current study, the rate of abnormalities in MRI was determined as 41.4%. In first recognized seizures, MRI abnormalities were reported as 31%. In patients with newly diagnosed epilepsies, 12.7% of abnormalities were demonstrated by Berg et al. using MRI. Amirsalari et al. reported 28.5% of abnormalities in MRI in epileptic children. This high rate of abnormalities detected using MRI may be due to the enrolment of patients having underlying neurologic conditions, such as cerebral palsy with new-onset seizures [8,25,26].

The EEG and MRI results were both abnormal in 87 (39.2%) patients. In five (2.3%) patients, both EEG and MRI results were normal. In five (2.3%) cases, EEG findings were normal and MRI results were abnormal. In a study by Doescher et al., 356 normal children with new-onset epilepsy were examined, and it was demonstrated that a normal EEG was not a reliable predictor of a normal MRI [27].

When an evaluation was performed according to the categories of epileptic discharges, the rate of abnormal MRI varied between 35.5% and 39.5% in cases with focal, generalized, and first focal to bilateral IED. No statistical significance was demonstrated. However, the presence of abnormalities in MRI was demonstrated to be significantly higher in the cases with multifocal IED (*p* = 0.04). Abnormal findings using MRI were detected in 68% of patients with multifocal IED. Zubcevic et al. demonstrated that 55.9% of the patients had bilateral IED in case of bilateral lesions observed by MRI, but clearly focal IED could also be observed [7]. Multifocal discharges in the present study were related to higher rates of abnormalities on the basis of the MRI findings.

Absence epilepsy is one of the epileptic syndromes that may occur in children that are otherwise normal. Among nine of patients, only one had abnormal findings on the basis of MRI examinations that incidentally detected a venous angioma. Although the sample size was too low for statistical analysis, this finding is consistent with those of previously published studies [8,24]. Images that were taken at 1.5 T demonstrated that there were no significant abnormalities in imaging in cases with typical clinical presentations, normal neurological examinations, and characteristic EEG findings. Those cases included benign epilepsy with centro-temporal spikes, childhood absence epilepsy, juvenile absence epilepsy, and juvenile myoclonic epilepsy [8,17,24].

When evaluations were performed according to discharges and MRI lesions, the results were difficult to interpret as the lesions were disperse. Berg et al. demonstrated that focal epileptiform discharges did not point out lesions in MRI [8].

Within the MRI findings, encephalomalacia, atrophy, hydrocephaly, and cysts were the most frequently observed pathologies in this study, detected in 15 (7.1%), 14 (6.6%), 9 (4.2%), and 9 (4.2%) patients, respectively. Amirsalari et al. reported abnormalities using MRI in 28.5% of the patients examined, and, in 10% of the cases, atrophy was the leading abnormality [26].

Additionally, in cases with bilateral brain lesions, like encephalomalacia, hydrocephaly, atrophy, gliosis, hypomyelination, and increased signal intensity, 27 (49%) out of 55 patients had generalized discharges. This was similar with the previous reports, that indicated 55.9% bilateral epileptiform discharges in cases with bilateral lesions evidenced by brain MRI. It was postulated that some of the findings detected by MRI are not the exact cause of epilepsy but are coincidental with other abnormalities, such as hypomyelination and delayed myelination often present in patients with metabolic disorders as a cause of epilepsy [7,27,28].

Within the study group of patients with a normal initial EEG, tumor formation was detected using MRI. The EEG discharges in case of space-occupying lesions may be normal, generalized, focal, or multifocal. In a study examining 59 patients with intracranial space-occupying lesion, scalp EEG was unlocalized in 29% of the cases, localized to the tumor area in 44% of the cases, and localized elsewhere in 27% of the cases [29].

## 5. Conclusions

A normal EEG does not rule out the presence of any pathology, including tumor formation. Thus, EEG findings are not a good predictor of MRI results. The presence of abnormalities in MRI was demonstrated to be significantly higher in cases with multifocal IED. Therefore, MRI examinations should be performed as soon as possible in cases with multifocal IED. This will be important for clinical diagnosis, treatment, and follow-up of epileptic children with multifocal IED.

## Figures and Tables

**Table 1:** Magnetic resonance imaging (MRI) and electroencephalography (EEG) findings classified as normal and abnormal.

MRI Results	EEG Normal	Discharge on EEG +	Total
MRI normal	5 (2.3%)	125 (56.3%)	130 (58.6%)
MRI abnormality +	5 (2.3%)	87 (39.2%)	92 (41.4%)
Total	10 (4.5%)	212 (95.5%)	222 (100%)

**Table 2:** Electroencephalography (EEG) findings in relation to the presence of abnormalities based on magnetic resonance imaging (MRI) readings.

MRI Results	No IED (*n*/total)	Focal IED	Generalized IED	Multifocal IED	3 Hz Spike and Wave IED	First Focal then Generalized IED	Total
Pathology according to MRI (number of cases/total)	5 (5/10)	19 (19/48)	39 (39/99)	17 (17/25)	1 (1/9)	11 (11/31)	92
Normal MRI (number of cases/total)	5 (5/10)	29 (29/48)	60 (60/99)	8 (8/25)	8 (8/9)	20 (20/31)	130
Total	10	48	99	25	9	31	222

IED: Interictal epileptiform discharges.

**Table 3:** Presence of abnormalities in magnetic resonance imaging (MRI) according to multifocal interictal epileptiform discharges (IED) (*p* = 0.004).

MRI Results		Multifocal IED		Total
		Present	Absent	
Abnormality on MRI	Present	17	70	87
	Absent	8	117	125
Total		25	187	212

**Table 4:** Electroencephalography (EEG) discharges in relation to magnetic resonance imaging (MRI) lesions.

MRI Lesion	No IED	Focal IED	Generalized IED	Multifocal IED	3 Hz Spike-Wave Discharges	First Focal then Generalized IED	Total (%)
Encephalomalacia	0	2	8	3	0	2	15 (16.3)
Hydrocephaly	0	1	4	2	0	2	9 (9.8)
Atrophy	0	4	4	4	0	2	14 (15.2)
Corpus callosum agenesis	0	1	1	0	0	0	2 (2.2)
Heterotrophy	0	2	2	1	0	0	5 (5.4)
Gliosis	0	2	4	0	0	1	7 (7.6)
Hypomyelinisation	0	1	4	0	0	1	6 (6.5)
Increased signal intensity	1	1	4	1	0	1	8 (8.7)
Megacisterna magna	0	1	0	0	0	0	1 (1.1)
Space-occupying lesions (tumor, cortical tuber)	1	1	1	1	0	0	4 (4.3)
Mesial Temporal Sclerosis	0	0	0	1	0	0	1 (1.1)
Cysts (cortical cyst, pineal cyst, arachnoid cyst)	2	1	4	2	0	0	9 (9.8)
Focal lesions (encephalomalacia, ischemic lesion, gliosis, hemorrhage)	1	2	1	2	0	2	8 (8.7)
Venous angiom	0	0	2	0	1	0	3 (3.2)
Total (%)	5 (5.4)	19 (20.7)	39 (42.4)	17 (18.5)	1 (1.1)	11 (12.0)	92 (100)
